# Beyond penile cancer, is there a role for sentinel node biopsy in urological malignancies?

**DOI:** 10.1007/s40336-016-0189-4

**Published:** 2016-07-04

**Authors:** O. R. Brouwer, H. G. van der Poel, R. F. Bevers, E. J. van Gennep, S. Horenblas

**Affiliations:** 1 Department of Urologyand Interventional Molecular Imaging Laboratory, Leiden University Medical Center, Albinusdreef 2, 2333 ZA Leiden, The Netherlands; 2 Interventional Molecular Imaging Laboratory, Department of Radiology, Leiden University Medical Center, Albinusdreef 2, 2333 ZA Leiden, The Netherlands; 3 Department of Urology, The Netherlands Cancer Institute—Antoni van Leeuwenhoek Hospital, Plesmanlaan 121, 1066 CX Amsterdam, The Netherlands

**Keywords:** Sentinel node, Urology, Urological malignancies, Penile cancer, Prostate cancer, Bladder, Renal cancer, Testicular cancer, Lymphatic drainage, SPECT/CT

## Abstract

This review aims to discuss the current state-of-the-art of sentinel node (SN) mapping in urological malignancies. The principles and methodological aspects of lymphatic mapping and SN biopsy in urological malignancies are reviewed. Literature search was restricted to English language. The references of the retrieved articles were examined to identify additional articles. The review also includes meta-analyses published in the past 5 years. SN biopsy for penile cancer is recommended by the European Association of Urology as the preferred staging tool for clinically node-negative patients with at least T1G2 tumours (level of evidence 2a, Grade B). The feasibility of SN biopsy in prostate cancer has been repeatedly demonstrated and its potential value is increasingly being recognised. However, conclusive prospective clinical data as well as consensus on methodology and patient selection are still lacking. For bladder, renal and testicular cancer, only few studies have been published, and concerns around high false-negative rates remain. Throughout the years, the uro-oncological field has portrayed a pivotal role in the development of the SN concept. Recent advances such as hybrid tracers and novel intraoperative detection tools such as fluorescence and portable gamma imaging will hopefully encourage prospectively designed clinical trials which can further substantiate the potential of the SN approach in becoming an integral part of staging in urological malignancies beyond penile cancer.

## Introduction

**Fig. 1:**
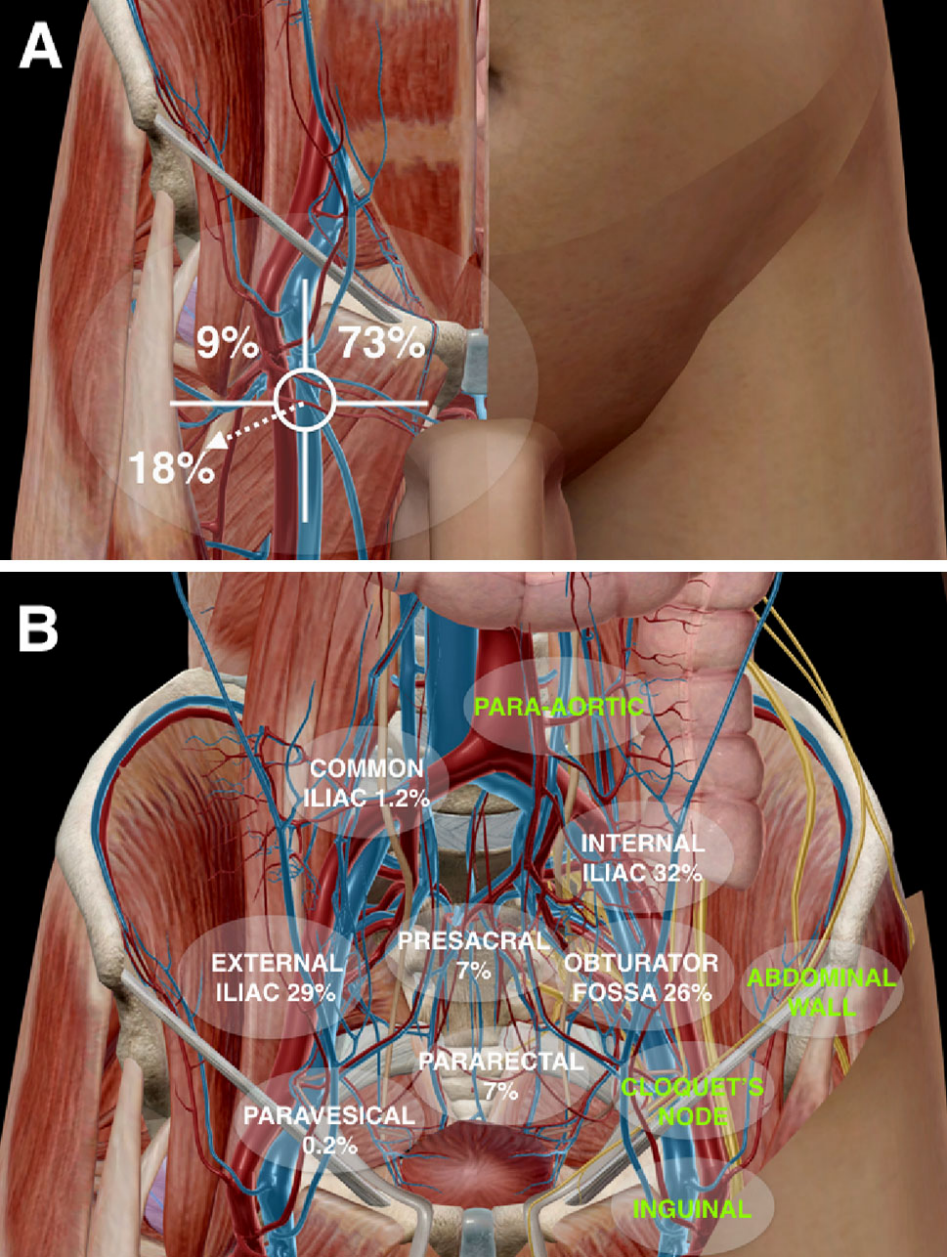
Anatomical sentinel node (SN) distribution. In penile cancer (**a**) SNs are limited to the groin and are predominantly found in the superior and central inguinal Daseler’s zones [28]. In contrast, prostate cancer (**b**) SN locations concern different pelvic basins (*white caption*) according to Wawroschek F et al. [65] as well as in other less common sites (*green captions*) according to Meinhardt W et al. (Prostate Cancer 2012;2012:751–3)

A sentinel node (SN) is defined as any lymph node on a direct drainage pathway from the primary tumour. This definition reflects the physiology of lymphatic drainage and the stepwise dissemination of cancer to a regional lymph node basin; it also acknowledges the possibility that more than one lymph node can be directly connected with the tumour and thus be a potential first site to harbour metastases before further progression to so called higher-tier/higher-echelon nodes [1]. With the introduction of the SN concept, a minimally invasive diagnostic modality emerged for early detection of occult lymph node metastases. Since its introduction more than 20 years ago for melanoma and breast cancer, the SN procedure has gone through a major development process and has become an essential component of lymph node staging in penile cancer [2, 3]. Consequently, this has led to an increasing interest in the application of the SN concept in other urological malignancies. Although both preoperative lymphatic mapping and intraoperative SN detection are common parts of the SN procedure for urological tumours, injection techniques and lymphatic drainage patterns may differ. In penile cancer, lymphatic drainage is mainly superficial and the first draining lymph nodes are usually located in the groin. In contrast, lymphatic drainage in prostate cancer and other urological tumours is deep and SNs are often found along the iliac vessels as well as in other complex anatomical areas (Fig. 1). This article covers the principles and methodological aspects of lymphatic mapping and SN biopsy in urological malignancies. The original introduction and evolution of the SN procedure in penile cancer is reviewed, as well as its potential role in prostate, bladder, renal and testicular cancer.

## Methods

An extensive literature search was performed based on the following PubMed/MEDLINE medical subject headings: SN biopsy, lymphatic mapping, SPECT/CT, intraoperative SN detection, hybrid tracer, penile cancer, prostate cancer, bladder cancer, testicular cancer and renal cell carcinoma. The search was restricted to English language. The references of the retrieved articles were examined to identify additional articles. Meta-analyses published in the past 5 years were also included.

### Penile cancer

#### Evolution and guideline recommendations

Penile cancer is a relatively rare disease in the western world, with an incidence of approximately 1 per 1,00,000 [4]. Penile cancer only rarely presents distant metastases without spreading via the lymphatic system to the inguinal regions first. As a consequence, the presence of lymph node involvement is the single most important prognostic factor for cancer-specific death [5]. At present, currently available non-invasive staging techniques lack sufficient accuracy to reliably stage the nodal status in patient with penile cancer which is why surgical staging remains indispensable. However, a complete inguinal lymphadenectomy is associated with substantial morbidity. Since only 20–25 % of clinically node negative patients harbour occult nodal metastasis, performing a complete lymphadenectomy is unnecessary overtreatment in 75–80 % of patients [6]. It is because of the abovementioned reasons that penile cancer has been an ideal model for the development of the SN concept. In 1977, it was the Paraguayan urologist Ramon Cabañas who was the first to publish a study using lymphangiograms to locate the primary draining lymph nodes followed by excision of these nodes [7]. In other words, Cabañas was the first investigator to perform lymphatic mapping followed by SN biopsy. However, Cabañas described the SN as to be localised in a fixed, predetermined location, not taking into account possible individual variations in lymphatic drainage patterns. Furthermore, Cabañas did neither use blue dye, radiotracers, lymphoscintigraphy nor a gamma probe, which made his method difficult to reproduce. Approximately 15 years later, the SN concept re-emerged outside the field of urology when Morton et al. performed SN biopsy in melanoma patients using radiocolloid and blue dye to intraoperatively visualise the lymphatic vessels and subsequently localise the SN [8]. This landmark study was the first to recognise variability in lymphatic drainage and SN location, which paved the way for further development of the approach. Based on this modernised concept, the SN procedure was re-introduced in uro-oncology in 1994 when Horenblas et al. initiated studies in the Netherlands applying the SN biopsy in its current form in penile cancer patients [9]. Since then, the procedure has evolved into a reliable staging technique, with a low complication rate compared to (prophylactic) inguinal lymphadenectomy [10–12].

The introduction of SN biopsy has also led to a significant improvement in mortality of patients with penile cancer. A recent study evaluated the 5-year cancer-specific survival of patients with squamous cell carcinoma of the penis. They compared the 5-year cancer survival rate before and after the introduction of SN biopsy and found a statistically significant difference, 91 % compared to 82 % (*p* = 0.021). This finding is most likely due to the detection of microscopic disease by SNB, resulting in early lymph node dissection in patients with a tumour-positive SN [13].

SN biopsy for penile cancer entered the European Association of Urology guidelines in 2009 as the preferred staging tool for clinically node-negative patients (defined by ultrasound-guided fine needle cytology) with at least T1G2 tumours (level of evidence 2a, Grade B) and the procedure is now standard of care in many (specialised) centres [3].

#### Methodological aspects

**Fig. 2:**
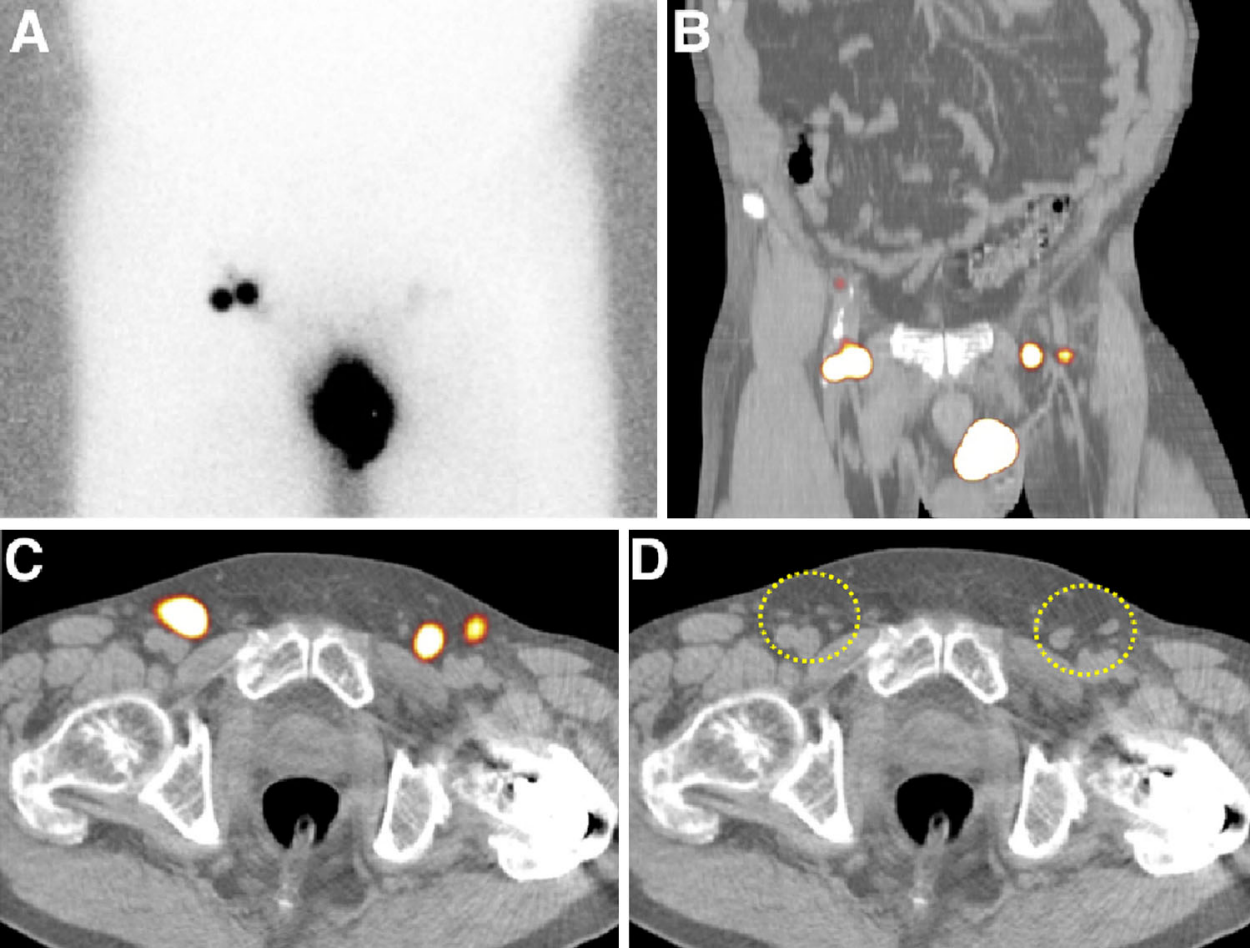
Lymphatic mapping in penile cancer. Planar anterior image **a** showing drainage to two lymph nodes in the right groin and faint left inguinal uptake. By contrast, coronal (**b**) and transversal (**c**) fused SPECT/CT show clear uptake in the left groin corresponding to two lymph nodes (*circle*) along both sides of the femoral vessels as seen on CT (**d**). Note that intense uptake in the right groin corresponds with a cluster of four lymph nodes (*circle*)

For penile cancer, there is a reasonable consensus regarding the preoperative lymphatic mapping procedure. The most widely applied radiocolloid (in Europe) is ^99m^Technetium-nanocolloid which is generally injected intradermally within 1 cm radius proximally from the primary tumour or from the surgical scar in case of prior primary lesion excision. A total tracer dose of 50–90 MBq in 0.2–0.4 cc volume divided in three sites is injected [14, 15]. The technique is also feasible after removal of the primary penile tumour by, e.g., partial penectomy (in this case the tracer is injected at the base of the penis), allowing for rapid removal of the primary tumour where needed and subsequent SN dissection in a specialised centre in a separate session [16]. Recently, this approach has successfully been applied in a series of 92 patients [17]. The evolution of the SN procedure throughout the years has led to a SN identification rate of 97 % with an acceptable false-negative rate of 7 % [18]. Repeat SN biopsy after tumour recurrence is also a validated procedure [19]. Lymphoscintigraphy after radiocolloid injection is mostly performed at 10–20 min and 2 h postinjection [12, 15]. The most frequently visualised lymphatic drainage pattern is bilateral drainage to both groins (80 %) and this technique has a reproducibility rate of 100 % [20]. In case of non-visualisation or unilateral drainage, tracer re-injection can be performed [21]. Some centres add single-photon emission computed tomography-computed tomography (SPECT/CT) to the imaging protocol to provide additional anatomical information of the SNs. For instance, the modality can differentiate inguinal from iliac (most frequently second-echelon) lymph nodes and is also helpful to detect additional SNs (Fig. 2) [22]. Furthermore, SPECT/CT has been used to optimise the procedure and to analyse the lymphatic drainage of penile cancer by evaluating the possible implications for the extent of inguinal lymph node dissection. In 50 patients lymphatic drainage was visualised in 82 of 86 clinically node-negative groins (95 %) scheduled for the SN procedure. All SNs were located in the inguinal zones (medial superior 73 %, lateral superior 8.7 % and central 18.3 % on SPECT/CT (Fig. 1a). No lymphatic drainage to the inferior zones of the groin was seen which suggests the possibility to exclude these zones from a subsequent inguinal lymph node dissection in the case of a tumour-positive SN [23].

As is customary for SN biopsy in melanoma and breast cancer, intraoperative SN detection is guided by a gamma-ray detection probe and blue dye. The entire (pre- and intraoperative) procedure can be performed in both 1- and 2-day protocols, although a 1-day protocol may yield lower false-negative rates [12]. Proper pathological node examination is imperative to minimise false-negative results. As stated in the EAU guidelines, additional inguinal node dissection is recommended in all men with nodal metastases at SN biopsy [3].

#### Refinements and future prospects

Initially, the most significant drawback of SN biopsy for penile cancer was found to be a relatively high false-negative rate (22 %) [24]. After analysis of the false-negative cases, several modifications were made to the procedure to decrease the false-negative rate and thus increase sensitivity: (1) histopathologic analysis was expanded with serial sectioning of the harvested SNs; (2) preoperative ultrasound of cN0 groins with fine needle aspiration cytology of suspicious lymph nodes was introduced and (3) exploration of the groin in the case of non-visualisation during scintigraphy was added, including intraoperative palpation of the wound to identify suspicious lymph nodes that failed to pick up any radiocolloid (due to tumour blockage) [25]. Thanks to these modifications, the procedure has evolved into a reliable minimally invasive staging technique, with an associated sensitivity of 93–95 % and low morbidity in experienced centres [10, 26]. However, higher false-negative rates have also been reported, a recent study stating a figure as high as 15 % and a later meta-analysis showing pooled sensitivity rates of 88 % [27, 28]. Accurate staging with SN biopsy can only be achieved if all nodes on a direct drainage pathway from the tumour are harvested. If SNs are left behind, this constitutes one of the potential causes for false-negative results. The following developments have refined the procedure and, in time, may help minimise false-negative rates. Preoperative imaging using SPECT/CT was shown to improve inguinal detection of SN by providing superior 3D information on lymph node location as compared to conventional scintigraphy imaging [22, 23, 25]. The integration of a portable gamma camera during the intraoperative procedure may also increase the detection sensitivity, since it provides an intraoperative overview image of the radioactive SNs and enables post-excision confirmation of complete removal of all SNs [29]. As mentioned before, the most widely used tracer for intraoperative optical detection of the SN and its afferent lymphatic duct is blue dye. The visualisation rate of lymph nodes with blue dye alone is between 55 and 70 % [29, 30]. In recent years, near-infared (NIR) imaging has become available as an additional optical guidance modality. Fluorescent tracers in the NIR spectrum of light provide the benefit of visualisation of lymph drainage without obscuring the surgical field in the white light surgical setting. Indocyanine green (ICG) is the most frequently used fluorescent dye that can be visualised using NIR imaging systems [31]. When injected locally, ICG rapidly migrates through the lymphatic system enabling intraoperative visualisation of lymphatic ducts and nodes, yet these quick migrational properties may also result in a limited diagnostic time frame and the visualisation of higher echelon nodes [32]. To solve this limitation, a recent development in penile cancer SN biopsy has been the introduction of a hybrid tracer molecule, comprised of the fluorescent dye ICG covalently attached to the ^99m^Tc nanocolloid [33]. This hybrid tracer molecule combines the properties of both modalities in one compound and can thus be used to provide both preoperative SPECT imaging and intraoperative fluorescence guidance during surgery [34]. The tracer demonstrated favourable characteristics compared to blue dye, allowing intraoperative visualisation of 96.8 % of SNs compared to blue dye which stained just 55.7 % [29].

### Prostate cancer

#### Concept and potential indications

Prostate cancer is the most frequent urogenital malignancy and the second most common cause of death by cancer in males [35]. Since prostate cancer may initially metastasize to the lymph nodes, lymph node staging is important for both prognosis and therapeutic management. For instance, the presence of lymph node metastases can disqualify patients from local therapy with curative intent, such as radical prostatectomy or external beam radiation therapy, and result in treatment with androgen-deprivation therapy instead [36]. Therefore, accurate lymph node staging in prostate cancer is imperative. To date, none of the available non-invasive diagnostic imaging modalities provide a reliable assessment of lymph node (micro) metastases; this is the principal reason why open or laparoscopic lymphadenectomy is still considered the gold standard for regional lymph node staging. Predictive nomograms are generally used to define the indication for a nodal dissection [37–40], and European guidelines recommend nodal dissection when nomograms predict a risk of more than 5 % on the presence of nodal metastases (intermediate and high-risk patients) [35, 37].

When performing a lymphadenectomy, an extended pelvic lymph node dissection template is advised, because dissection limited to the obturator fossa misses approximately 50 % of metastases [35]. Apart from its role in discriminating N0 from N1 patients, recent retrospective data suggest that an extended lymphadenectomy also has therapeutic value: in >300 pN1 prostate cancer patients, removal of less than 14 nodes resulted in poorer outcome when compared to removal of more lymph nodes [41]. On the other hand, an extended nodal dissection is associated with considerable morbidity, and complications of the procedure are strongly correlated with the number of nodes removed, ranging from 10.5 % for 1–5 lymph nodes to 24.3 % when dissection includes more than 20 lymph nodes [42].

Despite extended nodal dissection, pelvic recurrences do occur, and the false-negative rate of extended pelvic nodal dissection for prostate cancer was estimated to be around 12.5 % [43, 44]. This can potentially be explained by the finding that tumour-positive (sentinel) nodes may be present outside the extended pelvic node dissection template in >5 % of patients. With the aim to “remove the nodes that count, rather than count the nodes that don’t” [45], SN biopsy has emerged as an alternative staging procedure. The potential advantages of SN biopsy are a lower incidence of complications and the possibility of identifying tumour-draining lymph nodes outside the field of an extended lymphadenectomy [46]. If the goal is to remove as many (tumour-positive) nodes as possible to maximise a potential therapeutic effect, SN biopsy may be combined with an extended lymph node dissection [47, 48].

SN biopsy is generally reserved for patients in the intermediate-risk group (clinical stage T2b-c, PSA 10–20 ng/mL, Gleason 7) where it can identify lymph node metastases in 20 % of patients on average. Nevertheless, SN biopsy has been able to identify metastases in as many as 7–10 % of patients with more favourable risk factors [43]. In the intermediate-risk group, a pooled sensitivity rate of 95 % can be reached as reported by meta-analyses [49, 50]. The sensitivity appears to decrease to 76 % in high-risk patients [51].

As mentioned above, the presence of tumour-bearing lymph node(s) may influence the choice between local (curative) treatment of the prostate or hormonal therapy in patients in the intermediate-risk group. Another possible indication is to select patients who are eligible for salvage treatment of the prostate (in case of biochemical recurrence after local treatment). Selecting these patients is challenged by the fact that the usual parameters to stratify patients in risk groups do not apply to patients with intraprostatic recurrence. Since salvage treatment of the prostate may result in serious complications, it should only be considered when the prostate is actually the only tumour-bearing site [36].

At present, the European Association of Urology guidelines deem the SN procedure in prostate cancer at an experimental state due to lack of larger prospective studies and higher level of evidence. Nevertheless, guidelines acknowledge the potential role of the SN approach in defining lymph node dissection templates [35].

#### Methodological aspects

Tracer injection to identify SNs in prostate cancer is different compared to the technique used with other malignancies where the location of the primary tumour is often visible or palpable. In those cases, a well-directed peri- or intratumoural injection can be placed to observe the lymphatic drainage of the tumour. In prostate cancer, however, it is not known from which part of the organ the metastatic spread originates. Therefore, the aim of lymphatic mapping in prostate cancer has traditionally been to visualise all primarily draining lymph nodes (SNs).

Most studies use a transperineal or transrectal tracer injection method which is generally guided by ultrasound. One study described intraoperative optical tracer injection transperitoneally but in general, preoperative injection is used [52–54]. When the transrectal injection route is used prophylactic antibiotics are given to prevent infections. Injections are traditionally placed in both lobes in the peripheral zone of the prostate, since this is where most tumours (approximately 80 %) reside. However, tracer distribution may show considerable variation, which was recently demonstrated by Buckle et al. [55]. With the introduction of MRI-guided injection techniques, ongoing studies should reveal whether intratumoural injection is superior to several tracer depots within the entire prostate [56].

The most frequently used radiopharmaceutical in Europe has been ^99m^Tc-nanocolloid. However, technetium-99m can also be bound to sulphur or phytate as used in other continents [57, 58]. In literature studies particle sizes vary (80–1500 nm) as well as the amount of radioactivity injected (60–250 MBq) [59]. The particle concentration also appears to be important, and the use of a reduced labelling dilution volume (0.4 mL ^99m^Tc per 0.2 mg nanocolloid) yields more visualised SNs with higher radioactivity count rates [60]. No comparative studies are available on the optimal dose and injection volume as of yet.

Lymphoscintigraphy is mostly acquired 15 min after radiocolloid administration, which visualises the first draining lymph nodes in almost 88 % of cases [53]. Delayed imaging may be performed 2–4 h after injection (increasing the visualization rate to 90–95 %). Comparing the early and delayed images enables differentiation of second-echelon lymph nodes from SNs. Additional hybrid imaging with SPECT/CT enables anatomical localisation of SNs which can aid in planning the surgical procedure [44]. SPECT/CT was shown to increase the SN visualisation rate from 91 % for planar scintigraphy to 98 %. SPECT/CT also depicted more SN than planar images (average 4.3 versus 2.2 SNs) in 46 patients; 44 % of the SNs containing metastases were visualised only by SPECT/CT [61].

Intraoperatively, the radioactive SNs can be detected with a gamma-ray detection probe. Both open and laparoscopic approaches have been extensively studied in prostate cancer although there is a shift toward the less invasive laparoscopic and robot-assisted techniques [48, 62, 63].

#### Review of the results

The SN procedure for prostate cancer was introduced by the Augsburg group in 1999 [64]. SN biopsy was based on an open surgery approach with the use of a gamma probe to guide detection of the radioactive sentinel nodes. Initially, to evaluate the sensitivity of the procedure, an extended lymph node dissection was performed (common iliac, external iliac, obturator, internal iliac and presacral regions). After the initial feasibility report, the studied cohort was gradually expanded to a total of 638 patients, demonstrating an overall sensitivity of 97 % [65]. The most frequently occurring SN location was the internal iliac region (32 %), followed by the external iliac region (29 %) and obturator fossa (26 %, Fig. 1). The pioneering Augsburg group laid the groundwork for others to explore the SN approach for prostate cancer, reporting varying outcomes. In total, over 7000 cases were reported in the literature [66–68]. Overall, the median number of excised SNs was 6 (range 2–26) per patient. The SN detection rate ranged 76–100 % [46, 52, 54, 66, 67, 69–75]. In 4.1–25 % of patients, SNs outside the extended dissection template were found, whereas 3.5–17 % of patients with positive LNs had SN metastases outside the extended dissection template. In these studies, the median percentage of patients with positive LNs was 20.4 % (range 4.7–50) with a false-negative rate of 1 % (range 0–20), respectively, [44, 46, 52, 57, 64, 67, 70–82]. The largest study was performed by Holl et al. and published in 2009. Out of more than 2000 patients evaluated, only 11 false-negative cases (5.5 %) were reported [67]. A recent meta-analysis reported a pooled detection rate of 94 % (89–96.6 %) and a pooled sensitivity rate of 95 % (92–97 %) [49]. The potential advantages of the SN approach are a lower incidence of complications and the possibility of identifying tumour-draining lymph nodes outside the extended dissection field. Despite the relatively high sensitivity rates reported throughout the years, the routine use of SN biopsy in prostate cancer is still topic of debate as high level evidence from large prospective studies is still lacking. Recently, a large study by Abdollah et al. reported that removing a higher number of (tumour-positive) lymph nodes may improve cancer-specific survival rates [41]. This might support combining extended lymphadenectomy with SN mapping to prevent missing potentially relevant nodes which reside outside the extended dissection field.

#### Refinements and future prospects

Accurate localisation of SNs in the pelvis can be challenging. The introduction of SPECT/CT has refined preoperative lymphatic mapping by allowing exact pinpointing of the localisation of the SNs and providing anatomical reference points whilst planning the surgical procedure. SPECT/CT was shown to be able to identify SNs outside the extended dissection in 31 % of cases [46, 61]. The aberrantly located SNs were located proximal to the most distal part of the aorta, in the vicinity of the common iliac artery (above the crossing of the ureter), around the inferior mesenteric vessels, in the perivesical area and near the umbilical ligament.

**Fig. 3:**
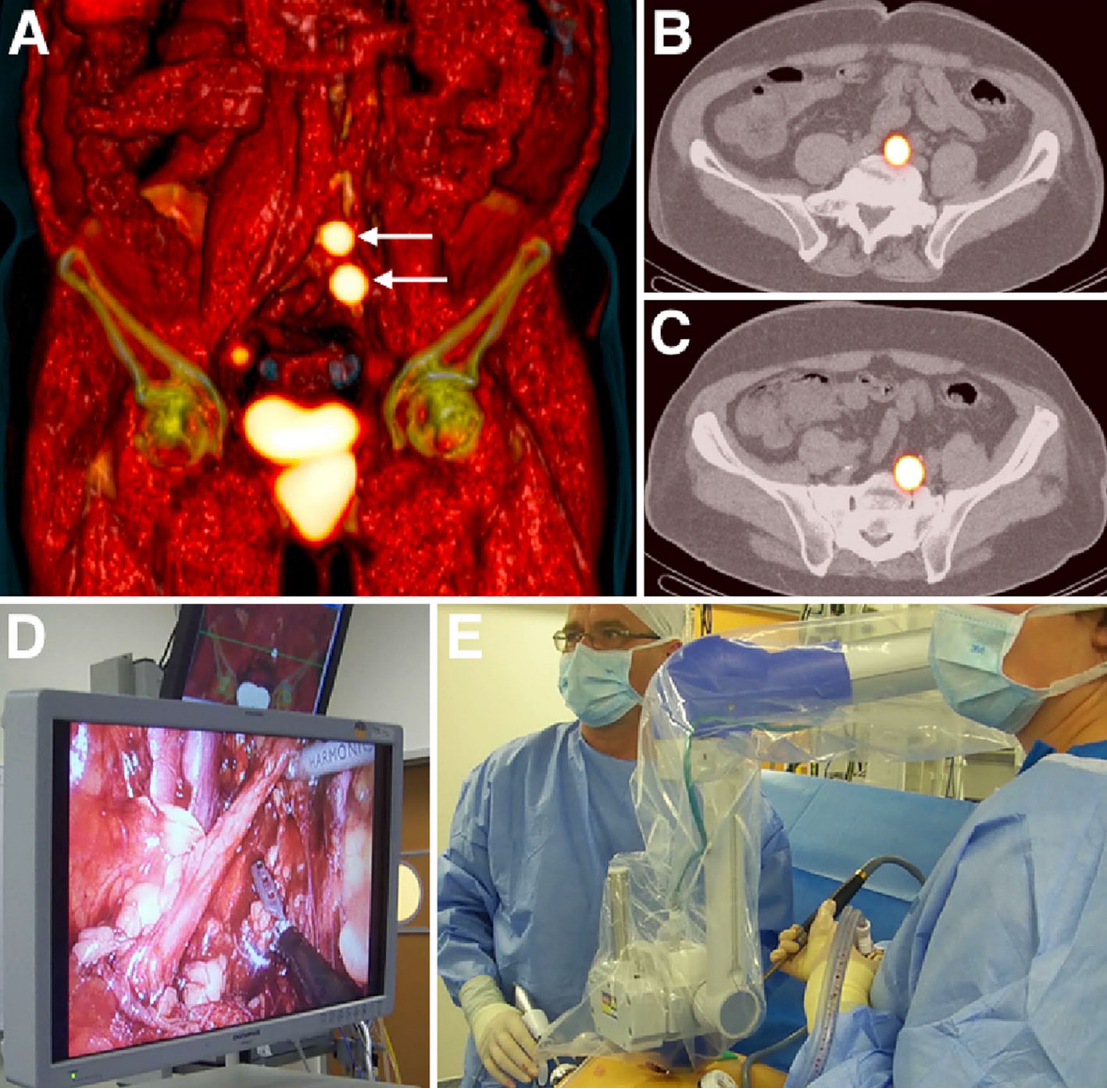
Sentinel node localisation in prostate cancer after administration of ^99m^Tc-nanocolloid. Coronal volume rendering (**a**) and transversal (**b**, **c**) SPECT/CT showing two sentinel nodes along the left common iliac artery. Based on this anatomical information both nodes are subsequently removed (**d**) laparoscopically, guided by a portable gamma camera (**e**)

Currently, the intraoperative procedure is generally carried out using a minimally invasive (laparoscopic and robot-assisted) approach. During laparoscopic surgery, the urologist traditionally localises an SN under guidance by the sound pitch originated by the laparoscopic gamma-probe. However, intraoperative spatial orientation using this device can sometimes be cumbersome, as a laparoscopic probe does not provide visual information. A particular innovation in the SN procedure for prostate cancer has been illustrated in recent years by the use of portable gamma to intraoperatively provide a two-dimensional image of the radioactive SNs (Fig. 3). The imaging system was shown to aid in confirmation of accurate SN removal in the laparoscopic setting [82, 83]. Current portable gamma cameras are capable of detecting two different signals: the signal of ^99m^Tc-nanocolloid for the visualisation of SNs, plus the signal of an iodine-125 (125I) seed pointer placed on the tip of the laparoscopic gamma-ray detection probe. The “hot” tip of the probe can be moved to the hot node, guided by the image of the portable camera (Fig. 3). This approach helps navigate towards the location of the SNs. Recent studies have also explored using the preoperatively acquired SPECT/CT images for intraoperative navigation (virtual/mixed reality) [84, 85].

**Fig. 4:**
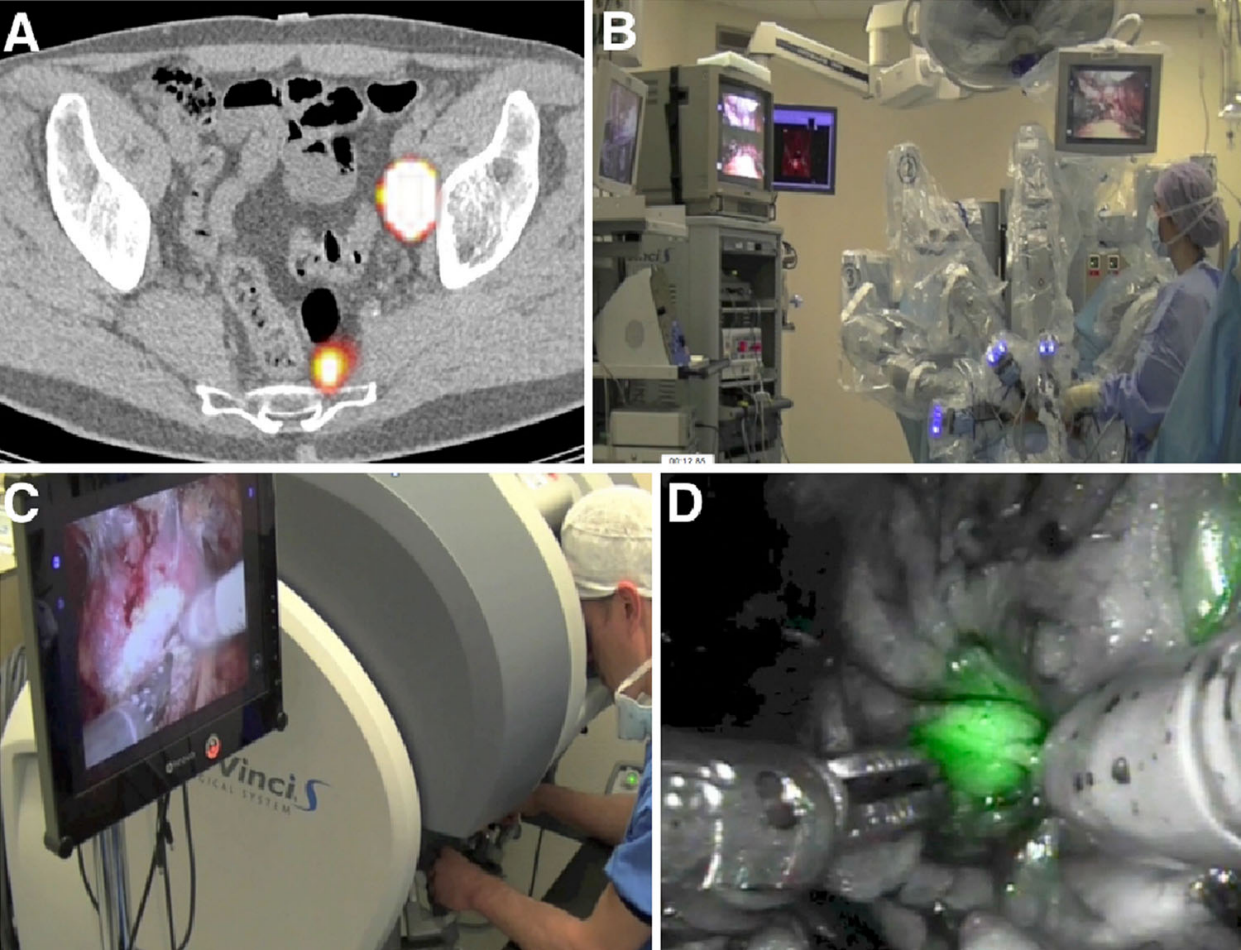
Following administration of the hybrid tracer ICG-^99m^Tc-nanocolloid in both lobes of the prostate, transversal SPECT/CT (**a**) shows iliac and presacral sentinel nodes, which are subsequently removed by means of a robot-assisted procedure (**b**–**d**)

In recent years, fluorescent dyes are increasingly being used during SN dissection in prostate cancer. These tracers are also injected intraprostatically and allow for realtime visualization of SNs and their afferent lymphatic vessels. One of the benefits of this approach can be that whereas intraoperative SN localisation using radiocolloid-based techniques can sometimes be hampered when SNs are located near the prostatic injection site (because of the high radioactive background signal), fluorescence-based techniques do not suffer from this limitation due to the relatively high resolution of fluorescence imaging systems. The most frequently used fluorescent dye has been ICG [86]. Using ICG in its free form has the benefit of rapid migration into the lymph nodes draining the prostate after intraprostatic injection [87]. Retention of tracer in lymph nodes, however, is much lower than with the use of nanocolloid bound ICG. Therefore, injection of free ICG is applied briefly (10–30 min) prior to the nodal dissection. This may provide logistical benefits compared to radiocolloid based tracing (no radioactivity, no preoperative scans), which may partially explain its increasing popularity [54]. However, although free ICG seems to provide a practical alternative gamma tracing, it does not allow for preoperative SPECT/CT and as a consequence, alternative lymph draining patterns may be missed due to the low penetrance of the NIR signal of ICG [88]. Studies using ICG bound to nanocolloid have also been performed and have shown to retain the properties of the original radiocolloid exhibiting longer retention times in the lymph nodes and allowing for combined pre- and perioperative imaging in open/laparoscopic procedures (Fig. 4) [34, 48, 75].

Another recent development is the use of ultrasmall superparamagnetic nanoparticle MRI [89–91]. A handheld magnetometer was used to detect intra-prostatically injected superparamagnetic iron oxide in 20 men with prostate cancer. Metastases were found only in lymph nodes detected using the magnetometer and identified as SNs in this analysis [91]. The role of this new approach for intraoperative detection of lymphatic drainage patterns holds promise but needs further evaluation.

### Other malignancies

#### Bladder cancer

In the United States, bladder cancer is the fourth most common type of cancer in men and the ninth most common cancer in women [92]. Lymph node metastases are common in patients with (muscle-invasive) bladder cancer, and the presence of lymph node metastases, their number and the volume of involved nodes are strongly associated with survival [93, 94]. Therefore, dissection of tumour-positive nodes may confer a survival benefit in patients with muscle-invasive bladder cancer with lymph node metastases, which is supported by studies comparing different historical cohorts indicating a therapeutic effect of extended lymphadenectomy [93, 95]. In addition, survival may also be increased by treating node-positive patients with systemic chemotherapy, in either a neoadjuvant or adjuvant setting [96]. These reasons underline the importance of accurate lymph node staging in bladder cancer patients. To date, the value of pelvic lymphadenectomy is undisputed for muscle-invasive bladder cancer. However, controversy exists on the extent of lymphadenectomy and the minimum number of nodes required for adequate lymphadenectomy, since lymph drainage from the bladder shows a wide variation and contralateral drainage is frequent [97–100] Moreover, single node metastasis may occur outside the known and accepted templates (e.g., along the common iliac and presacral areas) [100–102]. A LN mapping study using SPECT/CT after radiocolloid administration in 60 patients showed radioactive nodes in the external iliac (30 %), the obturator fossa (25 %), the internal iliac (26 %), the common iliac (15 %) and the para-aortic/paracaval (4 %) region [100]. This means that extended nodal dissection would have included 92 % of the active nodes, and a more limited resection of external and obturator template would have only included 50 % of the radioactive lymph nodes [100]. SN biopsy may provide a minimally invasive technique with the ability to identify potential tumour-positive nodes outside the field of extended lymphadenectomy. Interestingly, even extended lymphadenectomy followed by histologic examination may have its limits as approximately one-third of patients with pathologically staged pN0 bladder cancer show micro-metastases in LNs when molecular tools are used for staging [103–105]. Consequently, there might also be a role for SN mapping as a tool to improve nodal staging by selecting lymph nodes for ultrastaging, e.g., using serial sectioning and additional immunohistochemistry or reverse transcriptase (RT)‐PCR [106, 107]. Last, one report has proposed the use of SN biopsy to harvest T-cells for immunotherapy for bladder cancer [108].

The concept of SN biopsy was introduced to bladder cancer in 2001 [109]. Methodologically, various tracer injection techniques have been investigated, since both intraoperative and transurethral injection may yield different draining patterns [100, 110]. The recommended method is injection via a transurethral approach into the detrusor muscle, in four different sites adjacent to the visible bladder tumour [111]. In a recent study by Schaafsma et al. comparing the different injection techniques, cystoscopic injection with distension of the bladder also appeared to be optimal, allowing for detection of the SN in 11 out of 12 patients in this group (92 % detection rate). However, in this study only ICG was used and no preoperative lymphatic imaging was possible [112]. Most studies exploring SN mapping in bladder cancer used radioactive colloids and lymphoscintigraphy using a similar injection technique [100, 109, 113]. The use of radiocolloids also allows for preoperative SPECT/CT, which can provide a useful road map to guide urologists during the operation [114]. Intraoperative SN identification has mostly been performed in an open surgery setting guided by gamma-probes (and followed by extended pelvic lymphadenectomy). However, with the advent of minimally invasive (laparoscopic/robot-assisted) approaches, newer techniques for intraoperative SN detection such as the use of a portable gamma camera and NIR fluorescence imaging have also emerged [72, 82, 112, 115].

Taken together, the available studies report SN detection rates ranging from 81 to 92 %. Initial validation studies reported false-negative rates as high as 19 % [113]. These numbers form the basis of the persisting concerns around SN biopsy for bladder cancer as stated in the EAU guidelines [116], yet the numbers are comparable with the initial experiences in penile cancer, and more recent studies have shown higher sensitivity rates [105]. Nevertheless, the relatively high false-negative rates, the complexity of injection and additional need of an extended nodal dissection may explain the limited use of the SN procedure for bladder cancer management. Although SN biopsy for bladder cancer is still in the early development phase, new technologies such as portable gamma cameras, intraoperative navigation and hybrid/NIR fluorescent tracers may provide more insights into actual lymphatic drainage patterns and in turn, help reduce the number of false-negatives cases.

#### Renal cell cancer

Renal cell cancer (RCC) is the 8th most common malignancy in Europe and the incidence is rising [117]. The management of early stage RCC has traditionally been surgical, and nephrectomy or nephron-sparing strategies are often curative at this stage of the disease [118]. Even when patients present with metastases, nephrectomy and metastasectomy may be beneficial in selected cases [119, 120]. Metastatic spread has been shown to have a high correlation with tumour size and the widespread use of ultrasound examination of the abdomen and increasing use of whole-body screening (with CT or MRI) has led to more small renal tumours being diagnosed [121–123]. This stage shift may result in an increasing number of patients with early lymph node metastases, who, in contrast to historical data, may benefit from removal of these lesions [124, 125]. SN biopsy could have both a prognostic and therapeutic role in identifying and removing nodal disease in patients without distant metastases. Another reason for the interest in RCC SNs is the possibility within these nodes to harvest T-lymphocytes for preparation and reinjection for the novel approach of autologous adoptive immunotherapy [126]. In addition, the introduction of targeted agents has revived interest in adjuvant treatment concepts which also warrants accurate lymph node staging [127].

Since RCC has a well-recognised haematogenous spread and an unpredictable lymphatic drainage, the role of lymph node dissection in RCC remains controversial [124]. The general notion is that the draining lymph nodes from RCC are in the hilar region, branching off into the paracaval, inter-aortocaval or para-aortic retroperitoneal lymph nodes (depending on the tumour side). However, the lymphatic drainage of renal tumours may not always follow the known pattern, as has frequently been found for other tumour entities [128]. SN mapping offers the opportunity to identify such aberrant lymphatic drainage patterns in RCC.

**Fig. 5:**
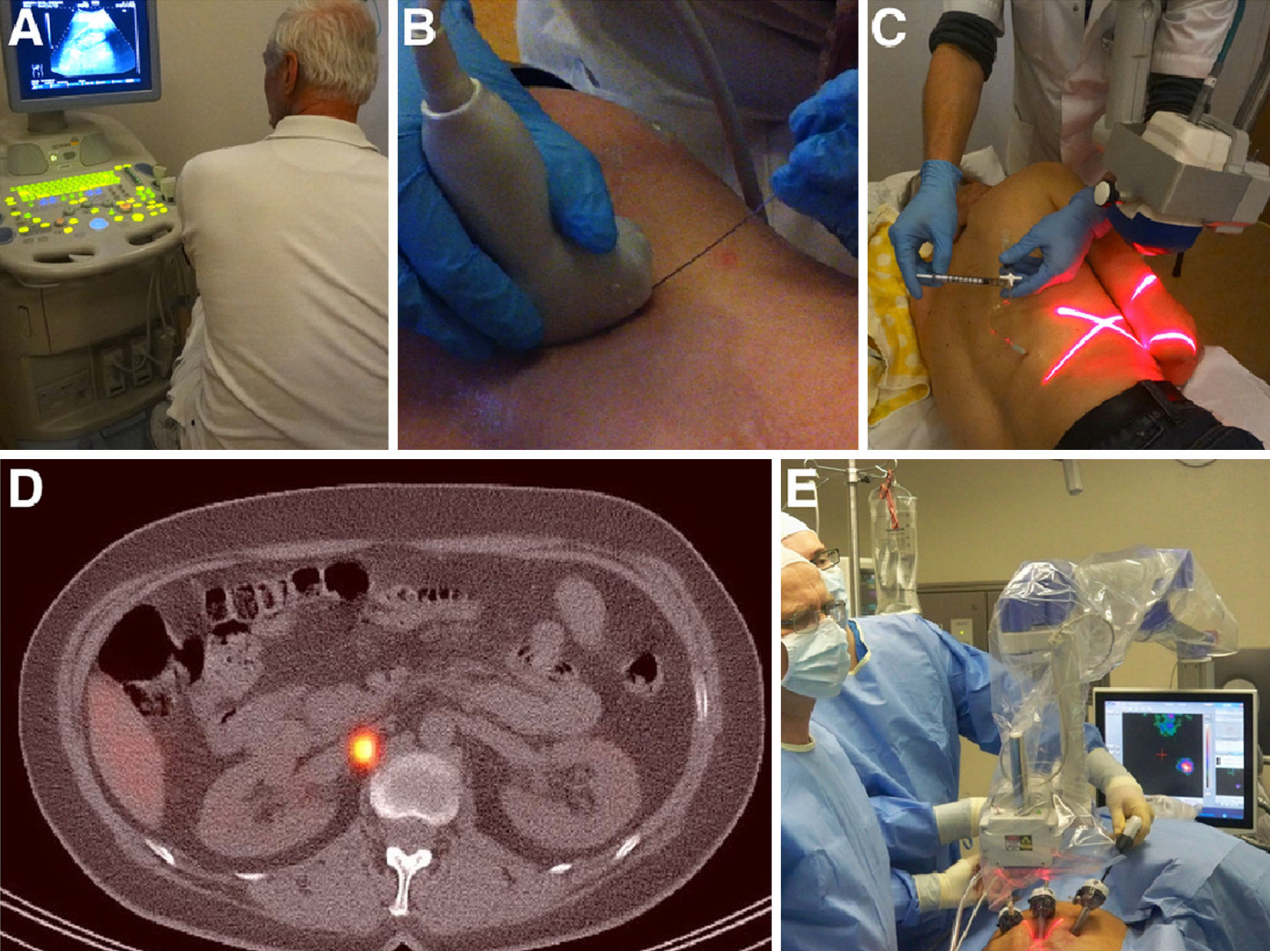
Sentinel node in renal-cell carcinoma. Following needle insertion under ultrasound guidance (**a**, **b**) ^99m^Tc-nanocolloid is administered into the tumour (**c**). Transversal SPECT/CT (**d**) shows a paracaval sentinel node, which is removed by means of laparoscopy assisted with a portable gamma camera (**e**)

After its initial feasibility was shown in porcine models by Bernie et al., the first pilot study exploring SN biopsy for RCC in humans was published by Bex et al. in 2010 [129, 130]. ^99m^Tc-nanocolloid was injected percutaneously into the renal tumour (<10 cm = cT1a/b-cT2a) guided by ultrasound or CT, followed by lymphoscintigraphy and SPECT/CT. The use of a portable gamma camera may assist tracer injection and help to modify/repeat the injection procedure in case radioactivity is detected outside the tumour and the kidney (Fig. 5). This approach led to successful SN identification in six of eight patients. Following a similar protocol, Sherif et al. detected 32 SNs in 10 of 11 patients in 2011 [126]. In a later follow-up study from Bex et al. expanding the cohort to 20 patients SN visualisation was possible in 70 % of cases. 2 of 20 patients had SNs outside the retroperitoneal region [131]. The absence of lymphatic drainage on imaging in 30 % of patients is of concern, in relation to a potential clinical application of this technique. This may be caused by lack of drainage of the radiocolloid through lymphatic vessels. Alternatively, the radiocolloid may have drained directly into the thoracic duct without any interposition of a lymph node, as has been proposed in a cadaver study by Assouad [132] and visualised in a recent study using SPECT/CT [133]. Intraoperative detection of SNs in RCC has been performed during open, and laparoscopic procedures and is generally carried out guided by a gamma-probe. Patent blue is not frequently used because of its limited contribution [126]. In the published series by Bex et al., one patient had two tumour-positive SNs at histopathology. The fact that all other excised lymph nodes during retroperitoneal lymph node dissection were tumour negative confirms the feasibility of SN procedure in RCC patients, although more extensive research is needed to further substantiate the diagnostic and therapeutic value of renal SN biopsy. Until then (extended) lymph node dissection still remains the management of choice in clinically node-positive patients without distant metastases [116].

#### Testicular cancer

Testicular cancer is the most frequent malignancy in young men, and the incidence has risen by almost 100 % in the past 20 years. At the time of diagnosis, approximately two-thirds of patients have clinical stage I disease [134]. The management of regional lymph nodes in stage I testicular cancer generally consists of a surveillance policy with intensive, frequent follow-up visits and costly examinations since occult metastases are present in less than 20 % of patients. The SN approach could potentially aid in selecting patients with lymph node metastases so that they can be treated at an early stage, while preventing unnecessary treatment for those patients without metastases. Furthermore, this could potentially save CT scans (and radiation exposure) for 80 % of patients with stage I cancer and reduce the number of patients maintained in follow-up.

**Fig. 6:**
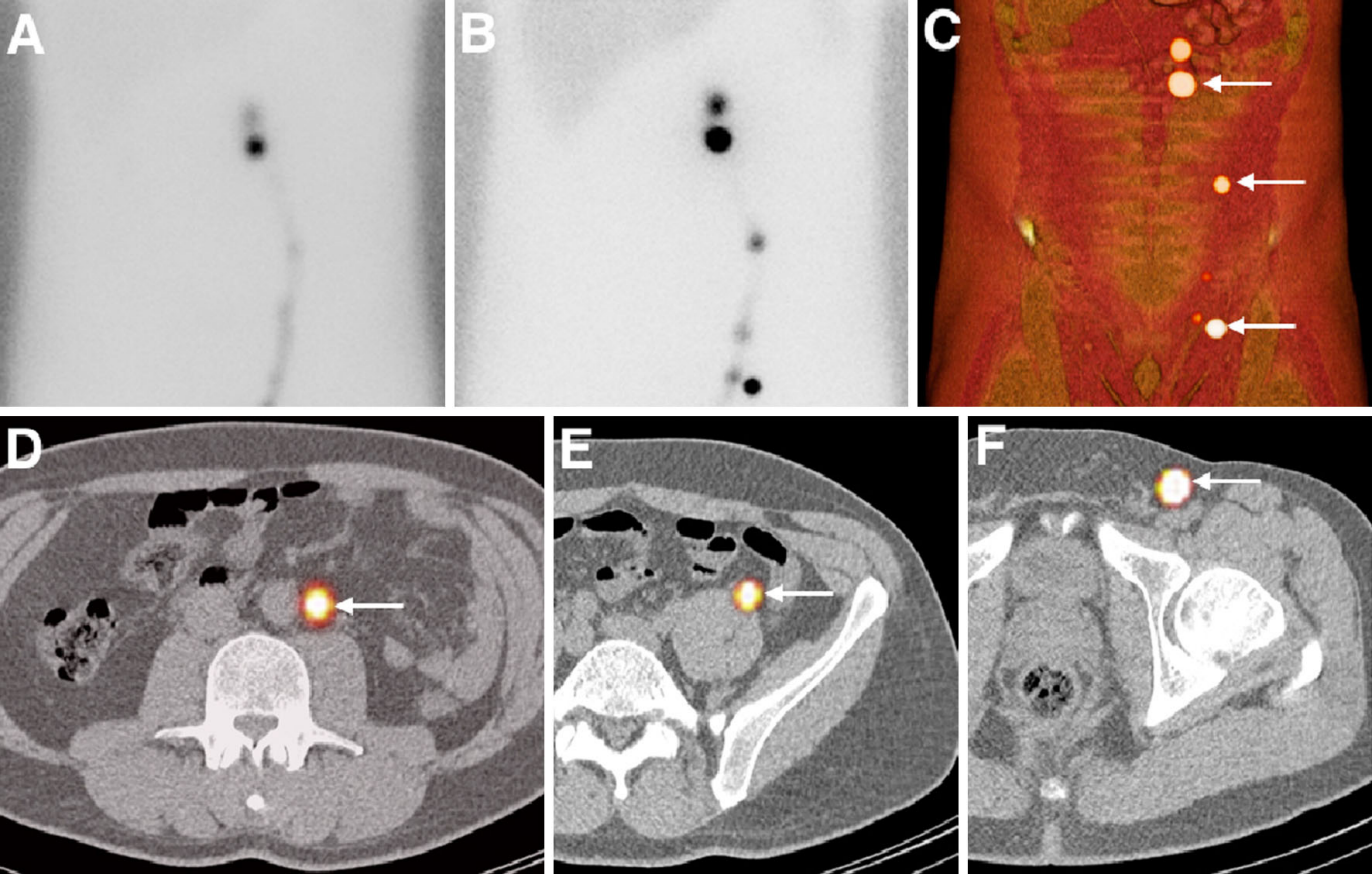
Lymphatic mapping in testicular cancer after injection of ^99m^Tc-nanocolloid in the left testicle. Early (**a**) and delayed (**b**) planar images showing lymphatic tract visualisation and uptake in various lymph nodes along the lymphatic duct. On SPECT/CT with volume rendering (**c**) three sentinel nodes are indicated (*arrows*) corresponding to localisations in para-aortic (**d**), funicular (**e**) and inguinal (**f**) basin

The use of SNs in testicular cancer is a relatively new concept and experience with the application is limited [135–138]. All published studies used radiocolloids as SN tracer. In the initial feasibility study by Tanis et al. in 2002, preoperative lymphatic mapping was performed using planar lymphoscintigraphy [135]. While funicular tracer administration showed only lymph node uptake in the inguinal region (which does not reflect the actual testicular tumour drainage pattern), intratesticular administration resulted in visualisation of retroperitoneal SNs in accordance with known drainage patterns. Following these footsteps, a group from Japan demonstrated a detection rate of 95 % in 22 stage I testicular cancer patients in 2005 [137]. Although lymphatic drainage of the testis is mainly directed towards the areas along the aorta and vena cava, aberrant drainage has also been observed [138]. The identification of these SNs in relation to the anatomical structures can be difficult using two-dimensional (2D) lymphoscintigraphy alone. SPECT/CT can provide useful anatomic information about the location of SNs and its improved sensitivity and added third dimension may also lead to the detection of additional SNs (Fig. 6). To date, one study evaluating the use of SPECT/CT for preoperative SN localisation in testicular cancer has been published. SPECT/CT enabled accurate localisation of the SNs and provided anatomical reference points to plan their laparoscopic retrieval [138].

In spite of the fact that SN biopsy for testicular cancer was shown to be feasible, large-scale randomised clinical studies to validate and assess the added benefit of the approach are still lacking. This may be partially due to the fact that patients are usually referred to tertiary/specialised centres after orchidectomy has already been performed, thus after removal of the potential injection site. More importantly, the need for improvement of staging in stage I testicular cancer is not pressing, with 5- and 10-year overall cancer-specific survival rates approaching 100 % [139]. On top of this, the use of adjuvant chemo and radiotherapy in case of suspected lymphatic dissemination at follow-up achieve very low recurrence rates and chemo-sensitivity among relapse patients is high, which is why SN biopsy does not currently have a role in lymph node mapping in testicular cancer in the EAU guidelines [139].

## Concluding Remarks

Throughout the years, the uro-oncological field has portrayed a pivotal role in the development of the SN concept, resulting in a well-established role of SN biopsy in penile cancer. Although its feasibility in prostate cancer has repeatedly been demonstrated and its potential value is increasingly being recognised, conclusive prospective clinical data as well as consensus on methodology and patient selection is still lacking. For bladder, renal and testicular cancer, only few studies have been published and concerns around high false-negative rates remain. Recent advances such as hybrid tracers and novel intraoperative detection tools such as fluorescence- and portable gamma imaging will hopefully encourage prospectively designed clinical trials which can substantiate the potential of the SN approach in becoming an integral part of staging in urological malignancies.
